# Use of peritoneal window and abdominal binder to reduce fluid collection after single-port robotic radical prostatectomy

**DOI:** 10.1007/s11701-025-02330-4

**Published:** 2025-04-12

**Authors:** Sonam Saxena, Mubashir S. Billah, Angelo Cadiente, Catherine Implicito, Daniel Thiemann, Sarah Brink, Aisha Kourouma, Ruth Sanchez de la Rosa, Michael D. Stifelman, Mutahar Ahmed

**Affiliations:** 1 Department of Urology, Hackensack University Medical Center, Hackensack, NJ USA; 2 Hackensack Meridian School of Medicine, Nutley, NJ USA

**Keywords:** Lymphocele, Radical prostatectomy, Peritoneal window, Single port, Robot-assisted

## Abstract

Fluid collections are common after robot-assisted radical prostatectomy (RARP) with pelvic lymph node dissection (PLND). Literature about surgical techniques to reduce fluid collections after extraperitoneal single-port (SP) RARP is limited. This study evaluates the proposed technique of bilateral peritoneal windows and abdominal binder to reduce postoperative fluid collections in extraperitoneal SP RARP. An analysis of extraperitoneal SP RARP cases (January 2019–May 2024) was conducted using IRB-approved retrospective and prospective databases. Two surgeons implemented the bilateral peritoneal windows plus abdominal binder intervention. Patients were divided into pre-intervention and post-intervention groups. Outcomes, demographics, and complications between groups were compared. One hundred ninety-five pre-intervention and 194 post-intervention SP RARP patients were analyzed. Post-intervention patients had higher BMI (28.9 kg/m^2 ^vs. 27.7 kg/m^2^, *p* = 0.018) and lymph node dissection rates (92.0% vs. 88.7%, *p* = 0.002), with shorter operating times (126.3 vs. 145.1 min, *p* < 0.001). Fluid collection rates decreased post-intervention (5.64% vs. 2.1%, *p* = 0.11), with fewer symptomatic cases (5.1% vs. 2.1%) and drainage requirements (2.0% vs. 1.54%). Non-fluid complications were significantly lower post-intervention during hospital stays (5.1% vs. 0%, *p* = 0.001). This study demonstrates that bilateral peritoneal windows and abdominal binder in extraperitoneal SP RARP may reduce incidence of postoperative fluid collection. The technique proved safe and efficient, with no bowel-related complications and shorter operating times. These findings highlight the efficacy of this approach and minimize patient burden due to this common complication.

## Introduction

Fluid collections are considered a common complication after radical prostatectomy (RP). Current literature varies in its expected rate of fluid collection after robot-assisted radical prostatectomy (RARP) and pelvic lymph node dissection (PLND), with estimated incidences ranging from 3.1 to 51% [1, 2]; symptomatic collections estimate 2.5–15.4% and are most often secondary to lymphoceles [1, 3].

Symptoms of fluid collections are secondary to compression of nearby structures which may lead to pain, infection, urinary symptoms, edema, and deep vein thrombosis [4, 5]. When symptomatic, many prefer drainage and, when appropriate, antibiotics. These consequences can be large burdens to the patient, emphasizing the need for techniques that minimize the incidence of these complications.

One potential technique for reducing postoperative fluid collections after extraperitoneal RARP is the creation of bilateral peritoneal windows, also known as peritoneal fenestration. This technique has been demonstrated in open RP literature; for example, Pose et al. found a significant reduction in symptomatic lymphoceles by adding a peritoneal window during open retropubic RP [6]; similarly, a randomized controlled trial found a reduced incidence of symptomatic and asymptomatic lymphoceles due to incorporation of peritoneal fenestration during endoscopic extraperitoneal RP with extended lymph node dissections (eLND) [7]. However, there remains a gap in literature describing this technique following extraperitoneal single-port (SP) robotic prostatectomy with or without standard lymph node dissection (sLND) patients.

Another potential reduction technique is placement of an abdominal binder to compress the potential space between the anterior bladder and abdominal wall. Current literature describes the utility of abdominal binder after hernia repair surgery [8] but there is a lack of literature describing use after prostatectomy. This study aims to help lay the foundation for its use.

Secondary to our early anecdotal experience and others suggesting that extraperitoneal radical prostatectomy increases the risk of fluid collections [9–11], we began to use the above two techniques to decrease the risk of fluid collections. This study will explore the clinical benefit of bilateral peritoneal windows as well as postoperative abdominal binder to reduce rate of fluid collection following extraperitoneal SP RARP, providing high-volume clinical experience.

## Methods

SP RARP cases at a tertiary-care center from January 2019 to May 2024 were collected in IRB-approved retrospective and prospective radical prostatectomy databases. Two surgeons began using the above two interventions to decrease fluid collection rate in May 2022. Patients were divided into pre-intervention (RARP before May 2022) and post-intervention (RARP after May 2022).

Peritoneal window technique was performed after urethrovesical anastomosis and completion of the pelvic lymph node dissection. An incision was created lateral to the median umbilical ligament, entering the peritoneum laterally. The incision was carried posteriorly to the iliacs and measured 4 cm or more. Abdominal binder was placed on each patient immediately after the procedure and was removed 48 h after. Placement of abdominal drains was surgeon-dependent and recorded. No drain was left greater than 23 h.

Consistent with other literature discussing post-RARP complications, these postoperative fluid collections were identified and diagnosed incidentally on postoperative imaging (including CT, MRI, or ultrasound) for another medical problem or found by imaging performed for postoperative symptoms.

We reviewed demographic and clinical data, including age at time of surgery, BMI, prostate size, operating time, surgical approach, lymph node dissection performed, number of nodes removed, and postoperative Clavien–Dindo complications. Of those with postoperative complications, we recorded symptoms at presentation, imaging performed and results of imaging, and treatment.

We considered symptomatic presentation of postoperative fluid collection to include symptoms of lower abdominal pain, flank pain, pelvic fullness, worsening frequency or urgency, and fever. Both asymptomatic and symptomatic fluid collections were included in analysis.

When a drain was placed, fluid was analyzed for creatinine, triglycerides, and bacteria. Patients with urinomas were excluded. Positive culture was defined as having 100,000 + organisms.

Fisher's exact test was used to evaluate differences between the two groups in terms of rates of collection, interventions required, fluid analysis, and complication rates. Two-tailed student’s t-test was performed to evaluate differences in demographic data. Statistical analyses were performed using SPSS (IBM, Armonk, NY). Results were considered statistically significant if they had a p value less than 0.05.

## Results

Overall, 195 pre-intervention and 194 post-intervention patients were collected. Results are summarized in Table 1.

**Table 1:** Demographic data, perioperative, and postoperative data compared between cohorts

	No peritoneal windows (n = 195)	Peritoneal windows (n = 194)	p value
Age, years, average (std)	61.8 (6.88)	62.13 (7.16)	p = 0.645
BMI, kg/m^2^, average (std)	27.74 (4.81)	28.88 (4.18)	**p = 0.018**
Prostate size, average (std)	38.94 (15.04)	41.42 (15.66)	p = 0.11
Operating time, minutes, average (std)	145.12 (53.1)	126.3 (38.77)	**p < 0.001**
% patients requiring LND	88.7%	92.0%	**p = 0.002**
Nodes examined, average (std)	6.16 (5.74)	4.34 (3.39)	**p = 0.0003**
Fluid collection rate	11 (5.64%)	4 (2.1%)	p = 0.111
Symptomatic collections	10 (5.1%)	4 (2.1%)	p = 0.172
Collections requiring drainage	4 (2.0%)	3 (1.54%)	p = 1.0
Location of fluid collection			
Lateral to bladder (region of LND)	8 (72.7%)	1 (25%)	p = 0.23
Anterior to bladder	2 (18.1%)	1 (25%)	p = 1.0
Prostatectomy bed	1 (9.1%)	2 (50%)	p = 0.15
Body fluid triglyceride, mg/dL average (std)	26 (10.55)	11.67 (4.04)	p = 0.067
% with positive fluid Culture	0 (0%)	1 (33%)	
Non-fluid collection hospital stay complications	10 (5.1%)	0 (0%)	**p = 0.001**
Non-fluid collection 30-day complications	8 (4.1%)	3 (1.5%)	p = 0.22

The bolded p-values are meant to demonstrate significant p-values found, which was defined as p<0.05

BMI (27.7 ± 4.81 kg/m^2^ vs 28.9 ± 4.18 kg/m^2^, *p* = 0.018) and number of LND procedures (88.7% vs 92.0%, *p* = 0.002) performed were higher in the post-intervention group. The majority of LND patients in both groups received standard templates (pre: 92.4%, post: 99.4%) with the average number of nodes removed being 6.16 (5.7) and 4.34 (3.4), respectively (*p* = 0.0003). Operating time (145.12 ± 53.1 min vs 126.3 ± 38.8 min, *p* < 0.001) was also lower in the post-intervention group.

After employing the peritoneal window plus abdominal binder intervention, there were clinically large decreases in rates of fluid collection (5.64% vs 2.1%, *p* = 0.11), symptomatic fluid collections (5.1% vs 2.1%, *p* = 0.172), and collections requiring drainage (2.0% vs 1.54%, *p* = 1.0), although these differences were not statistically significant.

Common presenting symptoms of symptomatic lymphoceles included groin swelling, groin pain, and fever. There were no incidences of thrombotic events within the fluid collection patients.

Post-intervention, there was a decrease in lymphoceles, fluid collections located lateral to the bladder in the regions of lymph node dissection (pre: 4.1% vs post: 0.5%) (Table 1). Collections anterior to the bladder and within the prostate bed, likely seromas, were similar in both cohorts (pre: 1.5% vs post: 1.5%).

Of the drains placed in the pre-intervention group, the average triglyceride count was 26 mg/dL (range 16–33) compared to the drains placed in the post-intervention group 11.67 mg/dL (range 7–14). None had a triglyceride count of > 187 mg/dl, a finding seen in patients with chylous ascites [12].

There was only one infected collection noted, which grew Gram-positive cocci in the post-intervention cohort and required oral antibiotics.

Post-intervention patients showed lower rates of non-fluid collection complications both during hospital stay (5.1% vs 0%, *p* = 0.001) and 30-day complications (4.1% vs 1.5%, *p* = 0.22).

## Discussion

Fluid collections, commonly lymphoceles and seromas, are a frequent complication of RARP, particularly the extraperitoneal approach. These collections result from lymphatic vessel damage during lymph node dissection and general tissue disruption, leading to fluid leakage into the surgical site [13]. Extraperitoneal RP cases are considered especially risky for postoperative fluid collection, as dissection occurs in the small, closed space of Retzius, unlike in transperitoneal cases where the large peritoneal cavity can resorb this excess fluid [7, 9, 10, 14, 15]. Fluid buildup can have significant clinical consequences, such as infections or thromboses, and necessitate further interventions, adding to patient burden.

While extraperitoneal RARP carries a higher risk of fluid collections, it also offers advantages like shorter operating times, reduced hospital stays, and fewer bowel-related complications [15, 16]. The use of SP robotic systems has made extraperitoneal RARP more accessible and reproducible by facilitating access and dissection within the confined retroperitoneal space. SP systems also allow for easier peritoneal window creation, potentially mitigating some fluid collection risks.

In our study, the symptomatic fluid collection rate in the pre-intervention extraperitoneal SP RARP patient cohort (5.1%) was in line with other studies (6%− 35.9%) [17, 18]. We sought to reduce this rate with an intervention that builds upon the foundation established by earlier research about reducing rates of fluid collections following RP.

### Current options for fluid collection prevention in extraperitoneal RARP

Early attempts to reduce fluid collections after extraperitoneal prostatectomy involved using the hemostatic matrix FloSeal (Baxter, Deerfield, IL, United States). A 2011 study showed promising but statistically insignificant results (*p* = 0.149) due to a small sample size, with lower lymphocele rates in the FloSeal group (FloSeal lymphocele rate: 3.1% vs non-FloSeal lymphocele rate: 14.5%) [19]. While per-patient treatment costs were lower with FloSeal, this was skewed by the single FloSeal lymphocele requiring only drainage, compared to more invasive interventions in the non-FloSeal group. Further research is needed to validate FloSeal's efficacy and explore other preventative strategies.

Peritoneal fenestration has been described for endoscopic extraperitoneal RP by Stolzenburg et al. [7]. This study demonstrated a significant reduction in (asymptomatic and symptomatic) lymphocele rates (6% vs 32%, *p* < 0.001). This study also showed that there was no difference in bowel complications between the fenestration group and the non-fenestration group. Our study corroborates the use of peritoneal fenestration in extraperitoneal RARP, but adds the utility of SP and demonstrates the way that SP facilitates and may improve this technique. In addition, the postoperative abdominal binder can compress the potential space between the anterior bladder and abdominal wall and leave little room for fluid collection. Our results did confirm this hypothesis by showing a > 50% decrease in incidence of fluid collections (overall and symptomatic) with a p value that approached significance (*p* = 0.11).

Our results highlighting the reduction in lateral fluid collections also agree with our reduction in lymphocele rate. Although there is no defined diagnostic criteria for lymphocele, as most studies rely on imaging and/or clinical picture to diagnose, it is likely that the lateral fluid collections were lymphoceles as they were located in the areas of lymphatic dissection. This is in contrast to the anterior or prostatectomy bed fluid collections, which were likely seromas forming due to fluid pooling in the potential space left by prostate removal.

### Safety and operative time benefits

We also demonstrated this to be a safe technique, as complications were lower in the peritoneal window and abdominal binder group (5.1% vs 0%, *p* = 0.001 for hospital stay complications, 4.1% vs 1.5%, *p* = 0.22 for 30-day complications). The post-intervention group did not have any instances of bowel-related complications such as postoperative hernia or small bowel obstruction. There was no extra time required when using this intervention; we demonstrated that average operating time was significantly reduced (145.12 min vs 126.3 min, *p* < 0.001) in the intervention group, which may be in part to surgeon learning experience. In addition, the use of the SP system allowed this intervention to be done more easily, given its dexterity and flexibility.

### Limitations

One possible limitation of this study is the simultaneous use of two new interventions, the abdominal binder and peritoneal fenestrations. Adoption of these two interventions makes it difficult to distinguish which intervention is more clinically significant in terms of reducing the incidence of fluid collection. We note that the current literature describing abdominal binder use has not shown a significant ability to decrease fluid collection formation. Another possible limitation of this study is that the pre-intervention group had a higher average nodal count (6.16 ± 5.74 nodes compared to 4.34 ± 3.39 nodes), which may be a surrogate for a more extensive dissection in this group; literature has suggested that the number of nodes is associated with the onset of a symptomatic lymphocele [20]. This study was also completed at a single-institution with high-volume SP surgeons, limiting the generalizability of the results. Finally, this study may not have been powered enough to find statistically significant differences; further study may explore this technique in multicenter and diverse practice settings.

## Conclusions

This is the first study, to our knowledge, demonstrating clinical success of the peritoneal window and abdominal binder technique in extraperitoneal SP RARP to reduce fluid collections. We show a greater than 50% reduction in incidence of fluid collection and symptomatic fluid collection and a decreased need for intervention, reducing burden to post-prostatectomy patients. This technique should be considered by any surgeon performing an SP extraperitoneal prostatectomy with lymph node dissection. Further multi-institutional study can increase generalizability of these results.

## Data Availability

No datasets were generated or analysed during the current study.
